# Cognitive and existential-motivational pathways from empathy to prosocial behavior: a parallel mediation study in a Chinese university sample

**DOI:** 10.3389/fpsyg.2026.1912571

**Published:** 2026-09-18

**Authors:** Wei Si, Pan Chen, Jie Cao, Yikun Zhong, Peng Lei

**Affiliations:** 1 School of Tourism and Cultural Industries, Sichuan Tourism University, Chengdu, China; 2 School of International Tourism and Culture, Guizhou Normal University, Guiyang, China; 3 School of Marxism, Southwest Petroleum University, Chengdu, China

**Keywords:** empathy, meaning in life, parallel mediation, prosocial behavior, self-efficacy

## Abstract

**Objective:**

This study examined a parallel mediation model in which empathy was hypothesized to be associated with prosocial behavior through two theoretically distinct pathways: a capability-oriented pathway via self-efficacy and an existential-motivational pathway via meaning in life, among Chinese university students.

**Methods:**

A cross-sectional survey was conducted with 1,549 university students (*M* age = 19.47 years, SD = 1.05, range 18–23; 71.7% female). Participants completed validated Chinese-language measures of empathy, self-efficacy, meaning in life, and prosocial behavior. Parallel multiple mediation analysis using PROCESS Model 4, adjusting for age and gender, with bootstrap confidence intervals (5,000 resamples) was employed to examine direct and indirect associations among variables.

**Results:**

Empathy showed a significant positive direct association with prosocial behavior (c′ = 0.175, *p* < 0.001). Both self-efficacy (indirect effect = 0.192, 95% CI [0.147, 0.238]) and meaning in life (indirect effect = 0.075, 95% CI [0.044, 0.110]) showed significant positive indirect associations with prosocial behavior. A formal bootstrap contrast indicated that the self-efficacy pathway was associated with a significantly larger indirect effect than the meaning-in-life pathway (contrast = 0.117, 95% CI [0.061, 0.173]). The overall model accounted for 37.9% of variance in prosocial behavior (*R*^2^ = 0.379). A supplementary analysis decomposing meaning in life indicated that this pathway was carried specifically by search for meaning rather than presence of meaning, an exploratory finding not anticipated *a priori*.

**Conclusion:**

These findings are consistent with a model of prosocial motivation in which empathy is associated with prosocial behavior through both a capability-oriented (self-efficacy) and a smaller, more tentative existential-motivational (meaning in life) indirect pathway. Given the cross-sectional design, these results should be interpreted as patterns of association rather than causal mechanisms; interventions incorporating both capability-building and meaning-making components alongside empathy training may offer complementary benefits for fostering prosocial engagement, pending validation through prospective intervention research.

## 1 Introduction

### 1.1 Background and significance

Prosocial behavior, defined as voluntary actions intended to benefit others (Eisenberg and Spinrad, 2014), is associated with individual well-being, community cohesion, and societal flourishing: it predicts enhanced psychological well-being, stronger social relationships, and greater life satisfaction (Aknin et al., 2013; Curry et al., 2018), and underlies the cooperative social systems through which communities address collective challenges (Penner et al., 2005), a pattern confirmed by large-scale meta-analyses (Hui et al., 2020; Li et al., 2026). Among the psychological factors associated with prosocial behavior, empathy is one of the most robust and consistent correlates across populations and contexts, with higher empathic concern linked to greater helping, volunteering, and altruistic responses to others’ distress (Batson, 2010; Eisenberg and Miller, 1987).

Despite this evidence, research on the empathy-prosocial behavior link has predominantly focused on direct associations or single-mediator models, limiting our understanding of the underlying psychological processes. Candidate mediators such as moral reasoning, emotional regulation, and social responsibility have typically been examined in isolation rather than within integrated frameworks accounting for multiple co-occurring mechanisms, with cognitive and affective factors often treated as competing rather than complementary accounts of prosocial motivation. Dual-pathway frameworks suggest that complex social behaviors may reflect the parallel operation of functionally distinct mechanisms (Evans, 2008; Kahneman, 2011), yet such frameworks have rarely been applied to empathy and prosocial action. The present study addresses this gap by examining a parallel mediation model in which two theoretically distinct mechanisms—one centered on capability beliefs and one on existential meaning—jointly account for the empathy-prosocial behavior association.

### 1.2 Empathy and prosocial behavior

Empathy, conceptualized as the capacity to understand and share others’ emotional experiences, encompasses both cognitive (perspective-taking) and affective (empathic concern) components that enable individuals to respond to others’ needs (Davis, 1994; Hoffman, 2000). Batson's (2010) empathy-altruism hypothesis offers a foundational account of this link, proposing that empathic concern sustains helping behavior even at personal cost, differing qualitatively from egoistic motives such as mood management or social approval—a proposition broadly supported by experimental research showing that conditions heightening empathic concern reliably increase helping even when egoistic motivations are minimized.

Meta-analytic and longitudinal evidence documents moderate to large effect sizes for this relationship across diverse behavioral forms and developmental periods, with empathy in childhood and adolescence prospectively predicting adult prosocial behavior (Eisenberg and Miller, 1987; Eisenberg et al., 1999; Mattan et al., 2016; Padilla-Walker and Carlo, 2014). Within Chinese university student populations specifically, empathy is a significant positive correlate of prosocial behavior across independent samples (Li et al., 2024; Xia et al., 2025), even after accounting for moral identity and sense of security (Peng et al., 2024), lending ecological validity to propositions developed primarily in Western contexts.

Considerably less attention, however, has been devoted to the specific pathways through which empathy relates to prosocial behavior. Two gaps remain: the role of capability beliefs—empathic individuals’ confidence in their ability to help effectively—has rarely been examined as a mediator, and whether empathic engagement shapes existential meaning, and whether this process independently relates to prosocial action, has received limited systematic attention. The present study addresses both gaps by testing self-efficacy and meaning in life as parallel mediating pathways.

*Hypothesis 1*: Empathy is positively associated with prosocial behavior.

### 1.3 Self-efficacy as a cognitive mediator

Although empathy may orient individuals toward prosocial behavior, whether this orientation is expressed in concrete helping action may depend on beliefs about one’s capacity to help effectively—a capability gap that existing accounts, which have extensively theorized the motivational and emotional dimensions of empathy, have left comparatively unaddressed. Self-efficacy, defined as beliefs about one’s capabilities to organize and execute courses of action required to produce given attainments (Bandura, 1997), is a theoretically well-grounded cognitive factor that may account for part of this translational process: Bandura’s social cognitive theory holds that behavioral intentions are more likely to be expressed in action when accompanied by confidence in relevant capabilities, such that empathic motivation without efficacy beliefs may be insufficient for prosocial behavior to occur. In the present study, this pathway is examined using a measure of general self-efficacy; we return to the distinction between general and helping-specific efficacy beliefs in the Discussion.

This rationale draws on research showing that perspective-taking, a core component of empathic processing, deepens understanding of others’ needs, circumstances, and feasible responses (Davis, 2018; Gehlbach and Mu, 2023), which may be associated with greater confidence in one’s capacity to address those needs; empathic engagement may also involve executive control processes, particularly cognitive flexibility in perspective shifting (Chiu and Yeh, 2018), which could plausibly reinforce efficacy beliefs in helping-relevant domains.

Empirical evidence is consistent with these propositions. Self-efficacy beliefs prospectively predict prosocial behavior even after accounting for stable personality characteristics (Caprara et al., 2010), and in Chinese university student samples, emotional regulation self-efficacy has been found to be positively associated with prosocial behavior, with meaning in life mediating this association (Li et al., 2023). Empathy-enhancing interventions incorporating active skill-building are associated with stronger prosocial outcomes than those emphasizing emotional resonance alone (Chua et al., 2021; Teding van Berkhout and Malouff, 2016), and self-efficacy beliefs account for unique variance in helping behavior beyond general prosocial motivation (Caprara and Steca, 2005). Collectively, these findings suggest that self-efficacy may represent a significant cognitive pathway linking empathy to prosocial behavior.

*Hypothesis 2*: Self-efficacy is positively associated with the relationship between empathy and prosocial behavior, such that empathy shows a significant indirect association with prosocial behavior through self-efficacy.

### 1.4 Meaning in life as an existential-motivational mediator

Alongside capability beliefs, a second theoretically distinct pathway may involve meaning in life—the sense that one’s existence has purpose, coherence, and significance (Steger, 2009). Whereas self-efficacy addresses whether one is capable of helping, meaning in life addresses the more existential question of why helping matters, a meaning gap that empirical models of empathy-driven prosocial behavior have left largely unaddressed. Frankl's (1963) logotherapy proposed that humans are fundamentally oriented toward the search for meaning, and that contributing to others’ welfare represents a primary source of existential significance, a framework contemporary research has extended by showing that meaning in life is positively associated with prosocial behavior through value-expressive mechanisms (Martela and Steger, 2016). Meaning in life, as operationalized in this literature, encompasses both an already-established sense of purpose (presence of meaning) and an active, ongoing search for such purpose (search for meaning), both of which have been theoretically linked to motivational and behavioral outcomes (Steger et al., 2006); the present study’s primary analyses examine meaning in life at the level of the total construct.

This proposed association draws on the existential insight that empathic engagement with others’ suffering may function as a meaning-making experience, heightening awareness of human interconnectedness and deepening individuals’ sense of life purpose as they perceive a clearer alignment between helping others and their own fundamental values (Martela and Steger, 2016). Recent empirical work directly supports this linkage: meaning in life significantly mediates the association between empathy for pain and prosocial behavior among Chinese nursing students (Xia et al., 2025), and functions as a meaningful mediator in broader social behavior models among Chinese college students (Li et al., 2023; Lyu, 2024), with convergent evidence among sports science students, where prosocial behavior predicted meaning in life and mediated the resilience-meaning link (Ulutürk et al., 2026).

This association is further grounded in self-determination theory’s account of purpose-driven motivation: behaviors aligned with personal values and life purpose are experienced as more intrinsically rewarding and persistent over time (Ryan and Deci, 2000), and individuals who experience helping as a meaningful expression of their core values report greater persistence in prosocial engagement (Weinstein and Ryan, 2010).

*Hypothesis 3*: Meaning in life is positively associated with the relationship between empathy and prosocial behavior, such that empathy shows a significant indirect association with prosocial behavior through meaning in life.

### 1.5 A parallel pathway framework for prosocial motivation

The parallel mediating structure proposed here draws loose conceptual inspiration from dual-pathway thinking in psychological research more broadly. Dual-system theories propose that cognition and behavior emerge from an analytic mode of deliberate, controlled processing and an intuitive mode of automatic, associative processing (Evans, 2008; Kahneman, 2011), a distinction that has also informed research on moral judgment (Greene et al., 2001; Haidt, 2001), though more recent work has raised important qualifications, arguing that evidence for dual-process accounts of moral judgment rests on largely untested assumptions and yields equivocal findings (Quinlan and Cohen, 2026) and proposing unified predictive-coding alternatives to structural dualism (Gim, 2025).

We emphasize that the present study does not test, and does not rely on, dual-process or dual-system theory in this formal sense: our design includes no manipulation of processing mode and no measurement of automaticity, response latency, or cognitive load. The term “parallel pathways” (and, where used descriptively, “dual-pathway”) refers only to the co-occurrence of two statistically independent mediators representing different content domains—capability beliefs and existential meaning—not different modes of cognitive processing. Self-efficacy is treated as a capability-oriented pathway, reflecting heightened beliefs about one’s competence to help; meaning in life is treated as a meaning-oriented pathway, reflecting a deepened sense of life purpose and value alignment, and the two are theorized to make independent contributions to prosocial behavior.

We modeled these two pathways as parallel rather than sequential for both theoretical and empirical reasons. Theoretically, capability appraisal and existential appraisal are conceptually distinct processes, and no established account specifies that one must necessarily precede the other. Empirically, although the two mediators were moderately intercorrelated in the present sample, this shared variance is consistent with partial conceptual overlap rather than a strict precursor relationship, and the cross-sectional design cannot adjudicate a specific sequential ordering even if one were proposed. A parallel specification therefore represents the more conservative and defensible modeling choice, allowing each pathway’s independent association with prosocial behavior to be estimated net of their shared variance.

Pathway-based models have recently been applied directly to prosocial behavior contexts, identifying moderators of when deliberative versus intuitive processing leads to ethical outcomes (Warner et al., 2024) and proposing a cognitive-energetic model linking parenting motivation to prosocial behavior (Liu et al., 2025). A model simultaneously examining capability beliefs and existential meaning as co-occurring, statistically independent mediators has, to our knowledge, received limited empirical attention in large samples.

*Hypothesis 4*: Self-efficacy and meaning in life serve as parallel mediating pathways in the association between empathy and prosocial behavior, with each pathway showing a significant and independent indirect association.

### 1.6 Theoretical model and research objectives

The theoretical framework developed here integrates insights from social cognitive theory (Bandura, 1997) and existential psychology (Frankl, 1963; Steger, 2009) to propose a parallel mediation model of how empathy relates to prosocial behavior. Rather than summarizing these frameworks in isolation, this integration generates a prediction that neither would produce alone: because capability beliefs are behaviorally proximal (directly concerned with executing helping actions) whereas existential meaning is more distal (concerned with helping’s broader significance), the two pathways may be expected to differ in relative magnitude, with the capability-oriented pathway plausibly exerting the stronger association. This comparative prediction—rather than either theoretical account alone, and unlike prior studies examining self-efficacy (e.g., Li et al., 2023) or meaning in life (e.g., Xia et al., 2025) as isolated mediators—constitutes a central contribution of this study, allowing the two pathways’ relative magnitudes to be directly and formally compared within a single model.

Figure 1 depicts empathy’s direct association with prosocial behavior alongside the two parallel indirect pathways described above. The present study tested this model in a large sample of Chinese university students, examining whether empathy shows significant indirect associations with prosocial behavior through both pathways simultaneously, and whether these differ significantly in magnitude. By providing an integrated empirical examination of these two pathways within a non-Western cultural context, this research contributes to the theoretical understanding of prosocial motivation and offers preliminary, theory-generating insights for future intervention research.

**Figure 1:**
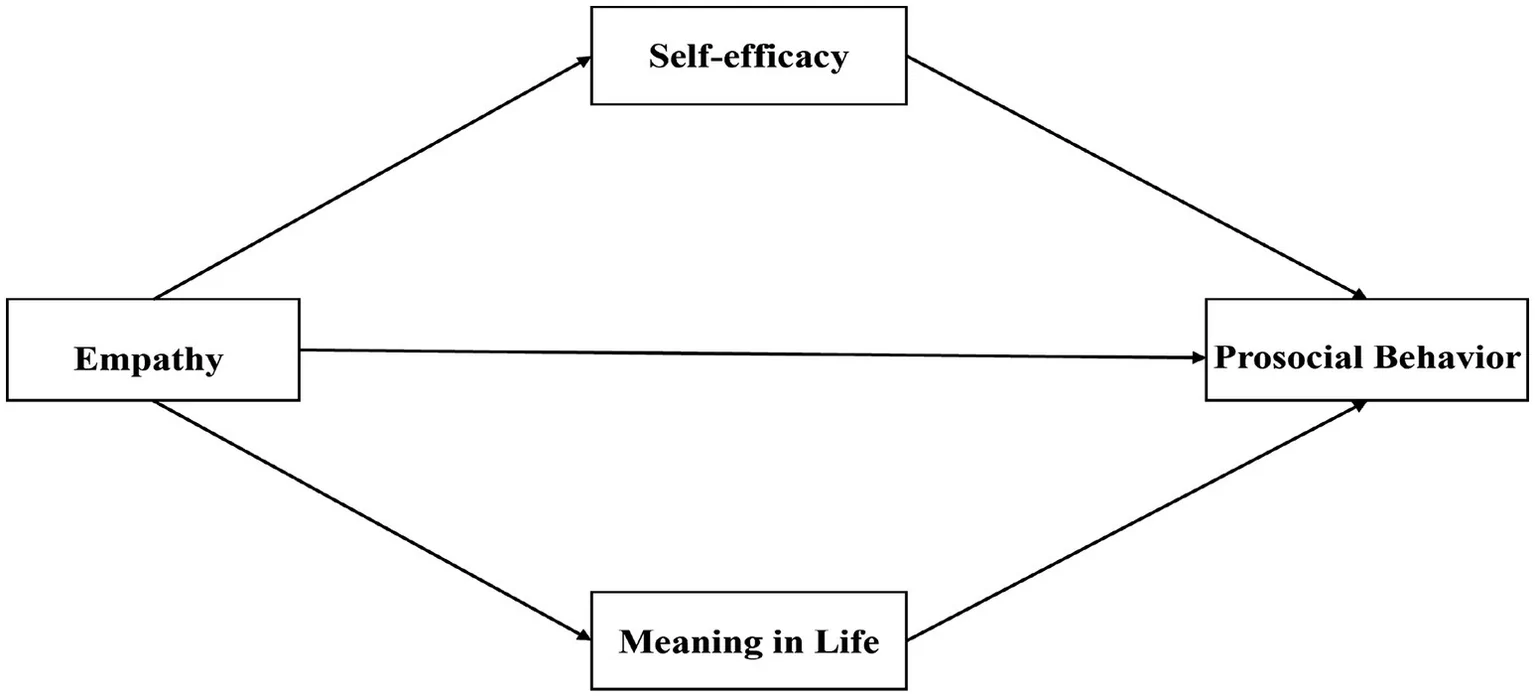
Theoretical conceptual model.

## 2 Methods

### 2.1 Participants

This study involved 1,549 Chinese university students recruited from six universities of varying institutional tiers in southwestern China, with questionnaires distributed at random within participating institutions and recruitment focused primarily on lower-year undergraduates. Aggregate participation counts were recorded at the institutional level; however, to protect participant anonymity, no individual-level institutional identifiers were collected, precluding record-level linkage between responses and specific institutions and, consequently, any formal evaluation of clustering effects (e.g., intraclass correlation) arising from shared institutional contexts.

The final sample consisted of 439 male (28.3%) and 1,110 female (71.7%) participants aged 18–23 years (*M* = 19.47, SD = 1.05), predominantly in the lower years of study (first-year: 53.2%, *n* = 825; second-year: 37.8%, *n* = 585; third-year: 7.8%, *n* = 121; fourth-year or graduate: 1.2%, *n* = 18). Participants were drawn from a range of broad disciplinary backgrounds spanning the humanities, arts, natural sciences, and social sciences; discipline-level (academic major) information was initially collected but, upon verification, was found to contain data-quality inconsistencies and was therefore excluded from both the demographic description and the covariate set—a data-quality-driven departure from our originally intended approach rather than an oversight in data collection. Most participants reported rural household registration (73.0%, *n* = 1,130) and modest parental education (61.3% of fathers and 68.0% of mothers had completed junior high school or below), and self-identified as Han Chinese (92.1%), with the remainder identifying as Yi, Qiang, Tibetan, or other ethnic groups (7.9% combined).

Of 1,600 respondents initially recruited, 21 were excluded for reporting ages inconsistent with the intended undergraduate sampling frame (suggesting postgraduate-student or teaching-staff status), and 30 were excluded for implausible response patterns, defined as an average per-item response time under one second or a total survey completion time exceeding 30 min, both indicative of inattentive or otherwise invalid responding. Attention-check items were additionally embedded throughout the survey to verify response engagement; however, no further exclusions arose from this screening, as all cases of inattentive responding had already been identified through the response-time criteria outlined above. These 51 exclusions in total yielded the final analytic sample of *N* = 1,549. Inclusion criteria required full-time undergraduate enrollment, generally in the lower years of study, age 18 or older, and sufficient Chinese language proficiency. All participants provided informed consent and received course credit or small monetary compensation.

### 2.2 Measures

#### 2.2.1 Empathy

Empathy was assessed using the Chinese version of the Interpersonal Reactivity Index (Davis, 1980; Chinese adaptation by Zhang et al., 2010), which measures four dimensions—perspective-taking, fantasy, empathic concern, and personal distress—via 22 items rated on a 5-point scale from 0 (does not describe me well) to 4 (describes me very well). Sample items include “I try to look at everybody’s side of a disagreement before I make a decision” (perspective-taking) and “I often have tender, concerned feelings for people less fortunate than me” (empathic concern). The Chinese adaptation has demonstrated adequate reliability and validity in Chinese populations (Zhang et al., 2010); in the current sample, the total scale showed excellent internal consistency (*α* = 0.941), with subscale reliabilities ranging from acceptable to good (perspective-taking *α* = 0.864; personal distress *α* = 0.848; fantasy *α* = 0.795; empathic concern *α* = 0.763). A confirmatory factor analysis of the four-factor structure, reported in the Results, indicated comparatively weaker fit than the other instruments, a limitation discussed further in the Limitations.

#### 2.2.2 Self-efficacy

General self-efficacy was measured using the Chinese version of the General Self-Efficacy Scale (Schwarzer and Jerusalem, 1995; Chinese adaptation by Zhang and Schwarzer, 1995), a 10-item measure of beliefs in one’s capability to manage demanding situations, rated on a 4-point scale from 1 (not at all true) to 4 (exactly true; e.g., “I can always manage to solve difficult problems if I try hard enough”). The scale has demonstrated strong psychometric properties across cultures (Scholz et al., 2002); internal consistency here was excellent (α = 0.951). We selected a general, rather than helping-specific, measure for its cross-cultural validation and established use in Chinese prosocial behavior research (Li et al., 2023), facilitating comparability with prior findings; we note, however, that general self-efficacy is conceptually broader than confidence specifically in one’s capacity to help others, a distinction revisited as a limitation in the Discussion.

#### 2.2.3 Meaning in life

Meaning in life was assessed using the Chinese version of the Meaning in Life Questionnaire (Steger et al., 2006; Chinese adaptation by Wang and Dai, 2008), a 10-item measure of presence of meaning (5 items, e.g., “My life has a clear sense of purpose”) and search for meaning (5 items, e.g., “I am looking for something that makes my life feel meaningful”), rated on a 7-point scale from 1 (absolutely untrue) to 7 (absolutely true). Its factor structure and construct validity have been supported in Chinese samples (Wang and Dai, 2008). The primary analyses used the total score (*α* = 0.928), consistent with our theoretical focus on global existential meaning as a unitary construct; because the two dimensions are conceptually distinct and have shown divergent associations with outcomes in prior research, we additionally examined them (presence *α* = 0.825; search *α* = 0.924) as separate parallel mediators in a supplementary analysis presented later in this article.

#### 2.2.4 Prosocial behavior

Prosocial behavior was measured using the Chinese version of the Prosocial Tendencies Measure (Carlo and Randall, 2002; Chinese revision by Kou et al., 2007), a 26-item scale assessing six helping types—altruistic, anonymous, dire (emergency), emotional, compliant, and public—rated on a 5-point scale from 1 (does not describe me at all) to 5 (describes me greatly; e.g., “I tend to help people who are in real crisis or need” [dire]). Its multidimensional structure has been validated across adolescents (Carlo et al., 2003). We computed a total score (*α* = 0.974), consistent with our focus on global prosocial engagement; subscale reliabilities ranged from good to excellent (altruistic *α* = 0.888; anonymous *α* = 0.896; dire *α* = 0.864; emotional *α* = 0.900; compliant *α* = 0.891; public *α* = 0.875). Zero-order correlations between each subscale and the study’s other core variables are reported in the Results and discussed in the Limitations.

Demographic information including age, gender, and family background was collected to characterize the sample and, where appropriate, to serve as covariates in the primary analyses.

### 2.3 Procedure

Data were collected via an online survey platform following approval from the Ethics Committee of the Sichuan Psychological Society (No. 2024033). Participants were recruited through course and student-organization announcements between April and May 2025, with materials stating the voluntary nature of participation; all reviewed an informed consent form detailing the study’s purpose, procedures, risks and benefits, confidentiality protections, and right to withdraw. To ensure data quality, attention-check items (e.g., “Please select ‘strongly agree’ for this item”) were embedded randomly within scales, and participants failing more than one were excluded; cases with an average per-item response time under one second or a total survey completion time exceeding 30 min were similarly flagged as indicators of invalid responding. These attention-check items were used solely for screening and were not retained as analytic variables.

Scale items were presented in random order within each questionnaire to minimize order effects, and participants completed the measures in a counterbalanced sequence, with empathy and prosocial behavior separated by the mediator measures and demographic questions; this procedural—rather than temporal—separation was intended to reduce the salience of the predictor-outcome relationship within the single survey administration. Participants received debriefing information upon completion, and all data were collected and anonymized in compliance with local privacy regulations and institutional ethical guidelines.

### 2.4 Statistical analysis

Analyses were conducted using SPSS 28.0 and the PROCESS macro (Hayes, 2018). Initial screening examined missing data, univariate and multivariate outliers (Mahalanobis distance), and assumptions including residual diagnostics, normality (skewness, kurtosis), and multicollinearity (VIF). Descriptive statistics and bivariate correlations were computed for all study variables, and confirmatory factor analyses evaluated each instrument’s hypothesized structure (fit indices reported in the Results).

Common method bias was formally evaluated using the unmeasured latent method construct (common latent factor) approach (Podsakoff et al., 2003, 2012), comparing a model with a method factor loading on all indicators against a baseline model without it, in terms of fit and the proportion of item-level variance attributable to method effects—a more rigorous alternative to the purely descriptive Harman single-factor test.

The primary analyses used parallel multiple mediation via PROCESS Model 4, with empathy as predictor, self-efficacy and meaning in life (total score) as parallel mediators, and prosocial behavior as outcome, adjusting for age and gender (academic major was excluded per Section 2.1). Significance of indirect effects was tested using 5,000-resample nonparametric bootstrap confidence intervals (MacKinnon et al., 2004), and both unstandardized and fully standardized coefficients are reported for all effects; standardized indirect effects are interpreted with reference to Cohen's (1988) conventions (0.01/0.09/0.25 for small/medium/large), reported alongside rather than in place of the effect estimates and confidence intervals themselves, as these benchmarks were not developed specifically for standardized indirect effects.

To formally test whether the two pathways differed in magnitude, we computed a bootstrap-based pairwise contrast between the self-efficacy and meaning-in-life indirect effects (Hayes, 2018). As a supplementary analysis, we re-estimated the model with meaning in life replaced by its presence and search subdimensions (entered alongside self-efficacy as a third mediator), computing pairwise contrasts among all three indirect effects. Sensitivity analyses re-estimated both models after excluding multivariate outliers to assess robustness. All analyses used an alpha level of 0.05.

## 3 Results

### 3.1 Descriptive statistics, measurement validation, and preliminary diagnostics

Descriptive statistics and reliabilities for the four core variables are presented in Table 1. Across the final sample of 1,549 Chinese university students, empathy (*M* = 47.214, SD = 13.293) and self-efficacy (*M* = 25.264, SD = 5.839) scores indicated moderate empathic responding and capability beliefs, while meaning in life (*M* = 44.472, SD = 8.688) and prosocial behavior (*M* = 89.515, SD = 16.572) scores suggested substantial engagement with existential purpose and helping behavior. All four measures showed excellent internal consistency (αs = 0.928–0.974; subscale reliabilities, αs = 0.763–0.924). Skewness and excess kurtosis fell within acceptable ranges for parametric analysis (empathy: 0.221/2.203; self-efficacy: 0.551/0.342; meaning in life: 0.326/2.791; prosocial behavior: 0.251/0.983; age: 0.588/0.258).

**Table 1:** Descriptive statistics and internal consistency reliability.

Variable	*k*	Scoring range	Observed range	M	SD	α
Empathy (IRI-C)	22	0–4	0–88	47.214	13.293	0.941
Self-efficacy (GSES)	10	1–4	10–40	25.264	5.839	0.951
Meaning in life (MLQ)	10	1–7	10–70	44.472	8.688	0.928
Prosocial behavior (PTM)	26	1–5	26–130	89.515	16.572	0.974

*N* = 1,549. IRI-C = Interpersonal Reactivity Index, Chinese revision; GSES = General Self-Efficacy Scale; MLQ = Meaning in Life Questionnaire; PTM = Prosocial Tendencies Measure.

#### 3.1.1 Preliminary data screening

No missing values were present on any focal variable or covariate. Univariate outlier screening (|*z*| > 3.29) identified 22 records, and multivariate screening via Mahalanobis distance [χ^2^(4) > 18.467, *p* < 0.001] identified 38 records; both were retained in the primary analyses, with their influence evaluated in a subsequent sensitivity analysis. No case exceeded conventional thresholds for influence (maximum Cook’s D = 0.063), residuals from the covariate-adjusted model showed acceptable distributional properties (skewness = 0.075, excess kurtosis = 2.056; 18 residuals exceeded |3|, range = −6.036 to 4.244), and VIFs (1.017–1.368) indicated no problematic multicollinearity.

#### 3.1.2 Measurement structure

Confirmatory factor analyses of each instrument’s hypothesized structure (Table 2) showed acceptable to good fit for the General Self-Efficacy Scale (one-factor) and Prosocial Tendencies Measure (six-factor), acceptable incremental fit but an elevated RMSEA for the Meaning in Life Questionnaire (two-factor), and comparatively weak fit across all indices for the four-factor Interpersonal Reactivity Index—Chinese revision, indicating that structural validity was not uniformly strong across instruments.

**Table 2:** Confirmatory factor analysis fit indices.

Model	χ^2^	df	χ^2^/df	CFI	TLI	RMSEA	SRMR
IRI-C four-factor	4961.75	203	24.44	0.766	0.734	0.123	0.098
GSES one-factor	468.51	35	13.39	0.966	0.956	0.089	0.029
MLQ two-factor	670.21	34	19.71	0.946	0.929	0.110	0.056
PTM six-factor	2419.79	284	8.52	0.939	0.930	0.070	0.035

*N* = 1,549. IRI-C = Interpersonal Reactivity Index, Chinese revision; GSES = General Self-Efficacy Scale; MLQ = Meaning in Life Questionnaire; PTM = Prosocial Tendencies Measure.

#### 3.1.3 Common method bias

Because all measures were self-reported within a single administration, common method bias was formally evaluated via the unmeasured latent method construct (CLF) approach (Podsakoff et al., 2003, 2012). Adding a single orthogonal method factor loading on all items substantially improved fit relative to a baseline model without it, Δχ^2^(68) = 4334.05, *p* < 0.001, ΔCFI = 0.051 (baseline: χ^2^(2132) = 12418.61, CFI = 0.877, TLI = 0.869, RMSEA = 0.056, SRMR = 0.062; with CLF: χ^2^(2064) = 8084.55, CFI = 0.928, TLI = 0.921, RMSEA = 0.043, SRMR = 0.033), with the method factor accounting for an average of 5.95% of item-level variance—smaller than would be expected if common method bias were a major driver of the associations reported below, though not fully excludable as a limited concern.

#### 3.1.4 Dimension-level associations

Because the primary analyses relied on total scores, we also examined zero-order correlations between each empathy subscale and prosocial behavior, and between each prosocial behavior subscale and empathy. All four empathy subscales correlated positively with prosocial behavior (*r*s = 0.272–0.384, all *p*s < 0.001), and all six prosocial behavior subscales correlated positively with empathy (*r*s = 0.300–0.343, all *p*s < 0.001), providing descriptive support for the use of aggregate scores.

### 3.2 Bivariate correlations among study variables

Bivariate correlations among the four core variables and the two Meaning in Life Questionnaire subdimensions are presented in Table 3. Empathy correlated positively with prosocial behavior (*r* = 0.354, *p* < 0.001, consistent with H1), with self-efficacy (*r* = 0.329, *p* < 0.001), and somewhat more strongly with meaning in life (*r* = 0.411, *p* < 0.001). Both mediators correlated positively with prosocial behavior (self-efficacy: *r* = 0.575; meaning in life: *r* = 0.406, both *p*s < 0.001) and with each other (*r* = 0.431, *p* < 0.001)—shared variance the parallel mediation framework statistically accounts for below. The presence and search subdimensions were highly correlated with each other (*r* = 0.799) and with the total score (*r*s = 0.941, 0.955), and showed comparable correlations with empathy (*r* = 0.425, 0.360) and prosocial behavior (*r* = 0.381, 0.390)—a similarity that, as shown below, diverged once both dimensions were entered simultaneously into the mediation model.

**Table 3:** Bivariate correlations among study variables.

Variable	1	2	3	4	5	6
1. Empathy	—					
2. Self-efficacy	0.329***	—				
3. Meaning in life (total)	0.411***	0.431***	—			
4. Prosocial behavior	0.354***	0.575***	0.406***	—		
5. Presence of meaning	0.425***	0.445***	0.941***	0.381***	—	
6. Search for meaning	0.360***	0.378***	0.955***	0.390***	0.799***	—

*N* = 1,549. ***p < 0.001.

### 3.3 Multiple mediation analysis

Parallel multiple mediation analysis was conducted using PROCESS Model 4 with 5,000-resample bootstrap confidence intervals, entering self-efficacy and meaning in life (total score) simultaneously so that each pathway’s indirect association was estimated net of the other. Because unadjusted and covariate-adjusted (age, gender) models yielded highly consistent results, the adjusted model is treated as primary, consistent with our analytic plan; full results appear in Table 4.

**Table 4:** Multiple mediation analysis results (parallel mediation model).

Path	Unadjusted *B* (SE)	*β*	Adjusted *B* (SE)	*β*	
Total effect (c)	0.442 [0.384, 0.500]	—	0.442 [0.383, 0.500]	—	
Direct effect (c′)	0.175 [0.120, 0.230]	—	0.175 [0.120, 0.229]	—	
a₁: Empathy → Self-efficacy	0.145 (0.011)***	0.329	0.145 (0.011)***	0.329	
a₂: Empathy → Meaning in life	0.269 (0.015)***	0.411	0.269 (0.015)***	0.412	
b₁: Self-efficacy → Prosocial behavior	1.319 (0.064)***	0.465	1.327 (0.064)***	0.467	
b₂: Meaning in life → Prosocial behavior	0.283 (0.045)***	0.148	0.280 (0.045)***	0.147	

Indirect/Contrast Effect	*B*	Boot SE	95% CI	Std. *B*	Std. 95% CI
Self-efficacy (unadjusted)	0.191	0.024	[0.146, 0.238]	0.153	[0.118, 0.189]
Meaning in life (unadjusted)	0.076	0.017	[0.045, 0.111]	0.061	[0.036, 0.089]
Contrast: SE − MLQ (unadjusted)	0.115	0.029	[0.058, 0.172]	0.092	[0.047, 0.136]
Self-efficacy (adjusted)	0.192	0.023	[0.147, 0.238]	0.154	[0.118, 0.190]
Meaning in life (adjusted)	0.075	0.017	[0.044, 0.110]	0.060	[0.035, 0.088]
Contrast: SE − MLQ (adjusted)	0.117	0.029	[0.061, 0.173]	0.094	[0.049, 0.139]

*N* = 1,549. Adjusted model controls for age and gender. Boot SE and 95% CI based on 5,000 bootstrap resamples. ***p < 0.001. Full covariate coefficients: Self-efficacy ~ Age, *B* = 0.129 (0.134), *p* = 0.336; Self-efficacy ~ Female, *B* = −0.578 (0.313), *p* = 0.065; Meaning in life ~ Age, *B* = 0.071 (0.193), *p* = 0.714; Meaning in life ~ Female, *B* = 0.455 (0.450), *p* = 0.313; Prosocial behavior ~ Age, *B* = −0.300 (0.319), *p* = 0.347; Prosocial behavior ~ Female, *B* = 1.140 (0.745), *p* = 0.126.

The total association between empathy and prosocial behavior was significant (c = 0.442, 95% CI [0.383, 0.500], consistent with H1), and remained significant though attenuated after including both mediators and covariates (c′ = 0.175, 95% CI [0.120, 0.229]), consistent with partial mediation.

Empathy was positively associated with self-efficacy (a₁ = 0.145, β = 0.329, *p* < 0.001), which was in turn positively associated with prosocial behavior after adjusting for meaning in life and covariates (b₁ = 1.327, *β* = 0.467, *p* < 0.001), yielding a significant indirect effect (0.192, 95% CI [0.147, 0.238]; standardized = 0.154, 95% CI [0.118, 0.190])—a medium effect by Cohen's (1988) benchmarks, consistent with H2.

Empathy was similarly positively associated with meaning in life (a₂ = 0.269, *β* = 0.412, *p* < 0.001), which was positively associated with prosocial behavior after adjusting for self-efficacy and covariates (b₂ = 0.280, *β* = 0.147, *p* < 0.001), yielding a significant but smaller indirect effect (0.075, 95% CI [0.044, 0.110]; standardized = 0.060, 95% CI [0.035, 0.088])—approximately a small effect, consistent with H3. Given the large sample size, this significance should not be equated with strong theoretical or practical importance; the effect is more appropriately characterized as modest and statistically reliable, warranting cautious interpretation.

A bootstrap-based pairwise contrast directly evaluating the two indirect effects was significant (self-efficacy minus meaning in life = 0.117, 95% CI [0.061, 0.173]; standardized = 0.094, 95% CI [0.049, 0.139]), formally confirming—rather than merely inferring from non-overlapping confidence intervals—that the self-efficacy pathway was associated with a significantly larger indirect effect, consistent with H4.

Neither age nor gender was significantly associated with self-efficacy, meaning in life, or prosocial behavior in the adjusted model (all *p*s > 0.06; Table 4). The adjusted model accounted for significant variance in prosocial behavior [*R*^2^ = 0.379, *F*(5, 1,543) = 188.00, *p* < 0.001], closely matching the unadjusted estimate [*R*^2^ = 0.377, *F*(3, 1,545) = 311.80, *p* < 0.001], indicating the substantive pattern was not attributable to the covariates (Figure 2).

**Figure 2:**
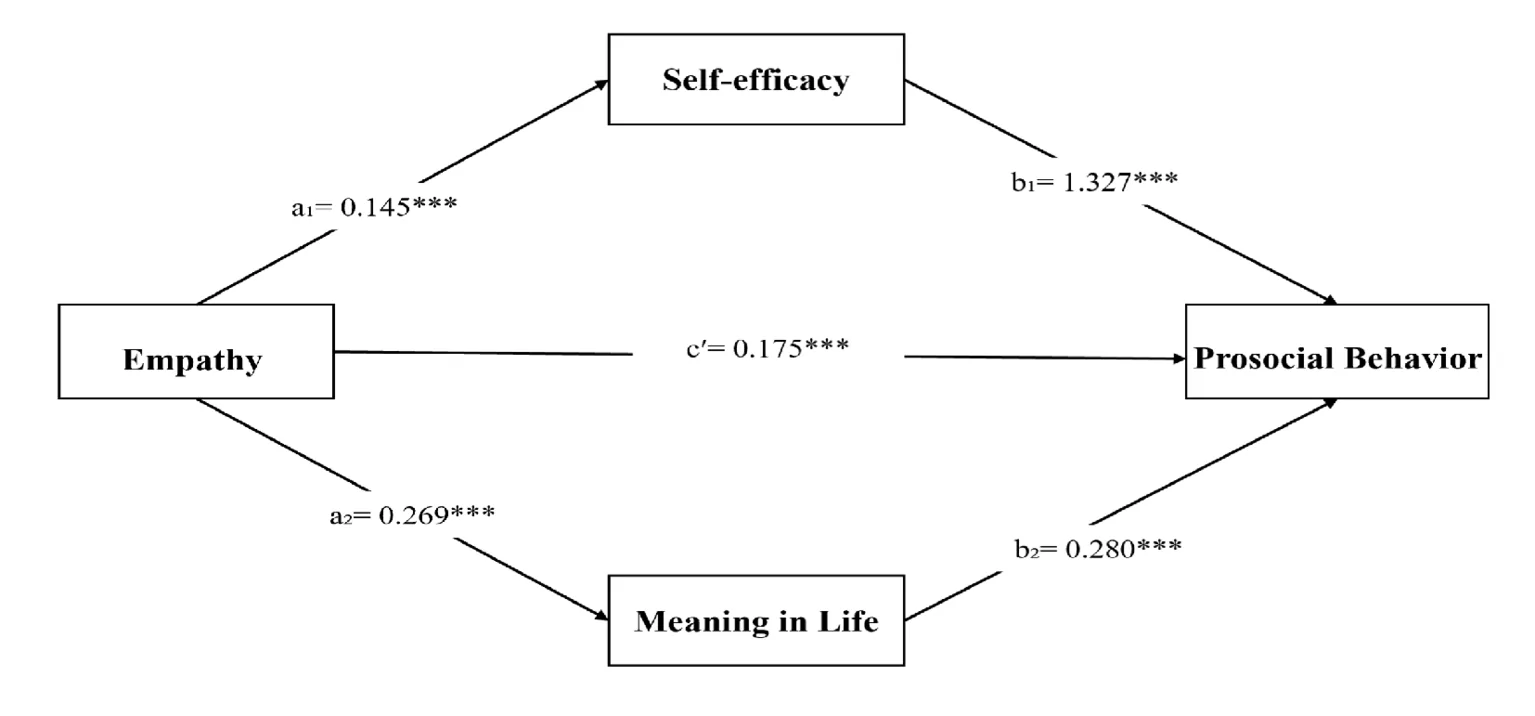
Path coefficients of the parallel mediation model. Values are unstandardized regression coefficients from the covariate-adjusted model (age and gender-controlled); standardized coefficients are reported in Table 4. All depicted paths are significant at ***p < 0.001. *R*^2^ = 0.379.

### 3.4 Supplementary analysis

Because presence and search for meaning are conceptually distinct and have shown divergent associations with outcomes in prior research (Steger et al., 2006), we re-estimated the model with meaning in life replaced by its two subdimensions, entered alongside self-efficacy as a third parallel mediator. This analysis was not part of our *a priori* hypotheses and is reported as an exploratory, hypothesis-generating extension.

In the covariate-adjusted three-mediator model [*R*^2^ = 0.384, *F*(6, 1,542) = 160.31, *p* < 0.001; unadjusted: *R*^2^ = 0.383, *F*(4, 1,544) = 239.47, *p* < 0.001], the self-efficacy pathway remained significant and comparable in magnitude to the primary model (0.196, 95% CI [0.151, 0.243]). Unexpectedly, the two meaning-in-life subdimensions diverged: presence of meaning showed a non-significant indirect effect (−0.028, 95% CI [−0.088, 0.033]), whereas search for meaning was significant (0.089, 95% CI [0.039, 0.145]). This pattern—search rather than presence accounting for the total-score pathway—was not anticipated by the theoretical framework outlined in the Introduction, which drew primarily on meaning as an already-established sense of purpose (e.g., Frankl, 1963; Steger, 2009); we report it transparently as an observed, exploratory result.

Formal contrasts confirmed the self-efficacy pathway was significantly larger than both the presence (0.223, 95% CI [0.149, 0.296]) and search (0.107, 95% CI [0.034, 0.179]) pathways. The presence-versus-search contrast was also significant in the primary sample (−0.116, 95% CI [−0.226, −0.013]); however, as shown below, this specific contrast did not survive the exclusion of multivariate outliers, whereas the significance pattern for each dimension separately did—so we report the presence-versus-search difference with corresponding caution (Table 5).

**Table 5:** Supplementary mediation analysis: meaning in life decomposed into presence and search (covariate-adjusted model).

Effect	Effect	Boot SE	95% CI	Std. effect	Std. 95% CI	Conclusion
Self-efficacy	0.196	0.023	[0.151, 0.243]	0.157	[0.122, 0.193]	Significant
Presence of meaning	−0.028	0.031	[−0.088, 0.033]	−0.022	[−0.070, 0.026]	Not significant
Search for meaning	0.089	0.027	[0.039, 0.145]	0.071	[0.032, 0.116]	Significant
Contrast: Self-efficacy − Presence	0.223	0.037	[0.149, 0.296]	—	—	Significant
Contrast: Self-efficacy − Search	0.107	0.037	[0.034, 0.179]	—	—	Significant
Contrast: Presence − Search	−0.116	0.056	[−0.226, −0.013]	—	—	Significant (not robust; see Section 3.5)

*N* = 1,549.

### 3.5 Sensitivity analysis

Both models were re-estimated after excluding the 38 multivariate outliers identified above (*N* = 1,511; Table 6). In the dual-pathway model, both indirect effects remained significant and, if anything, somewhat larger; in the decomposition model, the self-efficacy and search pathways remained significant and the presence pathway remained non-significant, but the presence-versus-search contrast, significant in the primary sample, was no longer significant. The core dual-pathway findings, and the basic pattern distinguishing a significant search pathway from a non-significant presence pathway, are thus robust to extreme observations, whereas the specific claim that search exceeds presence in magnitude should be treated as less robust.

**Table 6:** Sensitivity analysis: indirect effects after excluding multivariate outliers (*N* = 1,511).

Model	Effect	Estimate	95% CI	Conclusion
Dual-pathway	Self-efficacy	0.234	[0.190, 0.281]	Significant
Dual-pathway	Meaning in life	0.087	[0.059, 0.118]	Significant
Dual-pathway	Contrast: SE − MLQ	0.147	[0.092, 0.203]	Significant
MLQ decomposition	Self-efficacy	0.237	[0.194, 0.282]	Significant
MLQ decomposition	Presence of meaning	0.013	[−0.025, 0.055]	Not significant
MLQ decomposition	Search for meaning	0.065	[0.033, 0.100]	Significant
MLQ decomposition	Contrast: SE − Presence	0.224	[0.162, 0.287]	Significant
MLQ decomposition	Contrast: SE − Search	0.171	[0.119, 0.226]	Significant
MLQ decomposition	Contrast: Presence − Search	−0.052	[−0.119, 0.014]	Not significant

*N* = 1,511 (38 multivariate outliers excluded, Mahalanobis χ^2^(4) > 18.467, *p* < 0.001). SE = self-efficacy; MLQ = meaning in life.

## 4 Discussion

### 4.1 Summary of key findings

This study examined a parallel mediation model in which empathy was hypothesized to relate to prosocial behavior through two theoretically distinct indirect pathways—capability-oriented (self-efficacy) and existential-motivational (meaning in life)—among 1,549 Chinese university students. Findings supported all four hypotheses: empathy showed a significant direct association with prosocial behavior after accounting for both mediators and covariates (c′ = 0.175, 95% CI [0.120, 0.229]; H1); self-efficacy showed a significant indirect association (0.192, 95% CI [0.147, 0.238]; standardized = 0.154; H2); meaning in life showed a significant but comparatively smaller indirect association (0.075, 95% CI [0.044, 0.110]; standardized = 0.060; H3); and a formal bootstrap contrast confirmed these two associations differed significantly in magnitude (0.117, 95% CI [0.061, 0.173]), with self-efficacy the larger pathway (H4). The model accounted for approximately 38% of the variance in prosocial behavior (*R*^2^ = 0.379). A supplementary, exploratory analysis further indicated that this smaller pathway was carried specifically by search for meaning rather than presence of meaning—a pattern not anticipated by our theoretical framework and discussed below. These findings offer preliminary evidence for a model of prosocial motivation distinguishing capability-oriented from existential-motivational pathways, while underscoring that the two are not equivalent in magnitude.

### 4.2 Pathways to prosocial behavior: direct, capability-oriented, and existential-motivational associations

The direct association that persisted after accounting for both mediators is consistent with Batson's (2010) empathy-altruism hypothesis and prior meta-analytic evidence (Eisenberg and Miller, 1987; Mattan et al., 2016), and suggests, consistent with Hoffman's (2000) account of empathy as multifaceted, that the two measured mediators capture only part of the total association—a pattern further supported by findings that the empathy-prosocial behavior link persists after controlling for moral identity and sense of security (Peng et al., 2024).

The self-efficacy pathway, accounting for the majority of the indirect association, is consistent with Bandura's (1997) social cognitive theory and with the observed pattern (a₁ = 0.145; b₁ = 1.327 after adjusting for meaning in life). This aligns with the proposition that empathic perspective-taking is associated with greater confidence in addressing others’ needs (Davis, 2018; Gehlbach and Mu, 2023), and converges with longitudinal evidence linking self-efficacy to prosocial behavior (Caprara et al., 2010) and with Chinese evidence linking emotional regulation self-efficacy to prosocial behavior (Li et al., 2023). By formally comparing this pathway’s magnitude against a parallel existential-motivational pathway within a single model, the present study extends this literature beyond what prior single-mediator studies could establish.

The meaning-in-life pathway, though significant, was considerably smaller (standardized = 0.060, approximately a small effect); given the large sample, this significance should not be equated with strong theoretical or practical importance—a smaller or less representative sample might well have failed to detect it. With this caveat, the pattern (a₂ = 0.269; b₂ = 0.280 after adjusting for self-efficacy) is broadly consistent with the view that empathic engagement may be associated with a deepened sense of existential purpose (Frankl, 1963; Martela and Steger, 2016; Steger, 2009), converging with Chinese evidence (Xia et al., 2025; Li et al., 2023; Lyu, 2024) and with findings among sports science students (Ulutürk et al., 2026). The present cross-sectional data cannot establish whether this reflects a durable motivational foundation over time; longitudinal designs would be needed (Omoto and Snyder, 1995; Ryan and Deci, 2020).

Decomposing meaning in life into presence and search subdimensions revealed an unanticipated pattern: the indirect association was carried by search for meaning, which was significant, whereas presence of meaning was not. This pattern, though robust to the exclusion of multivariate outliers, should be distinguished from the specific claim that search exceeded presence in magnitude, which was not robust to that exclusion and warrants greater caution. We did not predict this pattern in advance and report it as an observed, hypothesis-generating finding. One speculative *post hoc* interpretation is that empathic engagement with others’ suffering may relate more closely to an active, exploratory orientation toward meaning than to an already-consolidated sense of purpose—searching for meaning may itself motivate prosocial engagement as one avenue through which meaning is pursued. We emphasize this as a direction for future confirmatory research rather than an established mechanism.

### 4.3 Theoretical contributions and cultural considerations

Integrating Bandura’s capability-based account with Frankl’s and Steger’s existential account generates a comparative prediction neither framework alone would produce: that a behaviorally proximal, capability-oriented pathway would exceed a more distal, existential-motivational one in magnitude—a prediction the present results support, extending rather than transforming existing frameworks. Within social cognitive theory, the empathy–self-efficacy association is consistent with perspective-taking enhancing helping-relevant confidence (Bandura, 1997; Davis, 2018; Gehlbach and Mu, 2023), a connection that has received limited systematic attention in this literature. Within existential-motivational theory, the empathy–meaning in life association offers preliminary support for empathic encounters as meaning-making experiences (Frankl, 1963; Martela and Steger, 2016; Steger, 2009), though the pathway’s smaller magnitude and its unexpected dimension-level composition indicate this account requires further refinement and replication. Whether these patterns are moderated by developmental, cultural, or contextual factors remains open, though a conceptual parallel exists in Liu et al.'s (2025) cognitive-energetics model.

The present sample and design offer only a limited basis for cultural interpretation. Chinese cultural traditions have been proposed to give constructs such as meaning in life a more relationally and collectively embedded character than in Western contexts (Li et al., 2024; Lyu, 2024), but as no measures of collectivism or relational ethics were collected, this interpretation is speculative and should be treated as a hypothesis for future cross-cultural research rather than a data-supported conclusion. The sample’s demographic composition further constrains any such inference (see Limitations), and the broader salience of culture as a boundary condition for prosocial behavior (Lin et al., 2025) underscores the value of future cross-cultural replication over *post hoc* explanation.

### 4.4 Implications for practice

The present findings carry preliminary implications for intervention design, though the cross-sectional design provides no direct evidence that manipulating self-efficacy or meaning in life would causally affect prosocial behavior; the following should be understood as theoretically derived hypotheses awaiting validation, not evidence-based guidance. With this caveat, the pattern observed—a larger capability-oriented and a smaller, more tentative existential-motivational association—suggests that programs might explore integrating both dimensions rather than relying on empathy-focused content alone (Chua et al., 2021; Durlak et al., 2011; Yu and Chou, 2018). Structured opportunities to build concrete helping skills (e.g., service-learning, collaborative problem-solving) may plausibly relate to the capability-oriented pathway, while brief reflective activities connecting helping to personal values (e.g., guided journaling) may plausibly relate to the existential-motivational one—though given its modest magnitude here, the latter’s practical value is considerably more uncertain and warrants direct evaluation before being prioritized.

Similar considerations apply in counseling and community settings: individual counseling might combine skill-building with brief exploration of clients’ values, and volunteer organizations might pair role training with reflection on how activities connect to personal meaning, rather than relying primarily on external incentives (Omoto and Snyder, 1995; Weinstein and Ryan, 2010). These are illustrative possibilities consistent with the present pattern, not validated intervention components.

Combined skill-building and meaning-making programs may prove more effective than either alone, particularly given the two pathways’ statistical independence here; however, this remains an empirical question for controlled intervention research—ideally comparing self-efficacy-focused, meaning-focused, and combined arms against an empathy-only control—rather than a settled basis for program design.

### 4.5 Limitations and future directions

Most fundamentally, the cross-sectional design precludes conclusions about temporal ordering or causality; findings should be interpreted as indirect associations rather than causal mechanisms, and alternative orderings cannot be ruled out (MacKinnon et al., 2004; Maxwell and Cole, 2007). Common method bias, formally evaluated via a latent-method-factor analysis, appeared modest (approximately 6% of item-level variance) relative to substantive factors but cannot be fully excluded, and the relatively high self-efficacy–prosocial behavior correlation (*r* = 0.575) warrants continued caution (Podsakoff et al., 2003, 2012; Richardson et al., 2009). Several measurement issues also merit attention. The four-factor empathy measure showed comparatively weak structural fit across all CFA indices (CFI = 0.766, RMSEA = 0.123), falling short of conventional thresholds; given this inadequacy in structural validity, the extent to which the IRI-C total score represents the intended empathy construct cannot be fully established, and the path coefficients involving empathy as a predictor should accordingly be interpreted with caution. Self-efficacy was assessed generally rather than in a helping-specific form; aggregate total scores were used for multidimensional empathy and prosocial behavior measures despite consistent subscale-level associations; and the exploratory search-versus-presence finding was neither anticipated theoretically nor fully robust across sensitivity checks. The sample—predominantly female, restricted to an 18–23-year age range reflecting our targeted sampling of lower-year undergraduates, and drawn from six universities of varying tiers in southwestern China—further limits generalizability: institutional identifiers were not collected (to protect anonymity), precluding evaluation of clustering effects; academic major was excluded after verification revealed data-quality inconsistencies; and cultural values such as collectivism were not directly assessed (Li et al., 2024).

Future research should employ longitudinal designs to evaluate whether these associations hold prospectively, and experimental designs with validated empathy-induction procedures and behavioral (rather than self-report) outcomes to strengthen causal inference and reduce method concerns. Multi-source designs incorporating observer-rated helping behavior and dimension-specific measures would clarify whether the present total-score associations mask more specific patterns. Cross-cultural replication directly assessing collectivism and retaining institutional identifiers for multilevel modeling is needed to evaluate the generalizability of both the relative pathway magnitudes and the search-versus-presence finding. Finally, intervention studies comparing self-efficacy-focused, meaning-focused, and combined programs would most directly test the possibilities raised above, and would clarify whether the modest meaning-in-life pathway reflects a genuine ceiling on its relevance or an artifact of the present measures and design.

## 5 Conclusion

The present study examined whether empathy’s association with prosocial behavior is accounted for by two theoretically distinct indirect pathways—a capability-oriented pathway via self-efficacy and an existential-motivational pathway via meaning in life—in a sample of 1,549 Chinese university students. The findings were consistent with this parallel mediation model: empathy showed significant positive indirect associations with prosocial behavior through both self-efficacy and meaning in life, each estimated independently after statistically adjusting for their shared variance, and a formal bootstrap contrast confirmed that the self-efficacy pathway was associated with a significantly larger indirect effect than the meaning-in-life pathway. A supplementary, exploratory analysis further indicated that this smaller existential-motivational pathway was carried specifically by search for meaning rather than presence of meaning, a pattern that was not anticipated by our original theoretical framework and that we report transparently as a direction for future confirmatory research rather than an established finding. Together, these results extend prior single-mediator accounts of the empathy-prosocial behavior association by suggesting that capability beliefs and existential purpose may each account for independent, though not equally sized, portions of this relationship, contributing incremental evidence for a more differentiated model of prosocial motivation within a Chinese university student context. The cross-sectional, self-report design limits causal inference, and replication using longitudinal, experimental, and multi-method approaches remains necessary before firm conclusions can be drawn. Pending such evidence, the present findings offer a theoretically grounded, hypothesis-generating basis for future research considering both capability-building and meaning-making components alongside empathy in educational and community programs aimed at fostering prosocial behavior.

## Data Availability

The raw data supporting the conclusions of this article will be made available by the authors, upon reasonable request.
